# Associations of coagulation parameters and thrombin generation potential with the incidence of type 2 diabetes: mediating role of glycoprotein acetylation

**DOI:** 10.1007/s10654-024-01162-0

**Published:** 2024-10-15

**Authors:** Jihee Han, Astrid van Hylckama Vlieg, Renée de Mutsert, Frits R. Rosendaal, Jeroen HPM van der Velde, Sebastiaan C. Boone, Esther Winters-van Eekelen, Saskia le Cessie, Ruifang Li-Gao

**Affiliations:** 1 Department of Clinical Epidemiology, C7-P, Leiden University Medical Center, PO Box 9600, Leiden, 2300 RC The Netherlands; 2 Department of Biomedical Data Sciences, Leiden University Medical Center, Leiden, The Netherlands

**Keywords:** Epidemiology, Coagulation factor, Thrombin generation potential, Type 2 diabetes, Glycoprotein acetylation, Mediation analysis

## Abstract

Hypercoagulability is characterized by abnormal elevations of coagulation factor levels and increased thrombin generation potential. Prior studies demonstrated links between impaired glucose metabolism, endothelial dysfunction, and hypercoagulability. However, the associations between hypercoagulability and incident type 2 diabetes as well as its underlying mechanism remain unclear. We aimed to assess the associations between coagulation parameters including coagulation factor (F) VIII, FIX, FXI, fibrinogen, thrombin generation potential (lag time, endogenous thrombin potential [ETP], peak, time-to-peak, velocity) and incident type 2 diabetes, and to study the underlying mechanism by examining the mediating role of glycoprotein acetylation (GlycA). In the Netherlands Epidemiology of Obesity study, we applied a Cox Proportional-Hazards Model in 5718 participants after adjustment for confounders. We further conducted a mediation analysis investigating the mediation effect of GlycA on the observed associations. During a median follow-up of 6.7 years, 281 incident type 2 diabetes diagnoses were reported. Compared with the lowest quartile, hazard ratio (95% confidence interval) of the highest quartile was 2.47 (1.48–4.14) for FIX, 1.37 (0.85–2.20) for FVIII, 1.11 (0.76–1.63) for FXI, 0.98 (0.65–1.48) for fibrinogen, 1.56 (1.07–2.28) for ETP, 1.84 (1.23–2.74) for peak, 1.59 (1.08–2.33) for velocity, 0.92 (0.62–1.38) for lag time, and 1.21 (0.86–1.70) for time-to-peak. GlycA mediated only a small proportion of all observed associations. In conclusion, elevated levels of coagulation factor and thrombin generation potential are associated with incident type 2 diabetes, suggesting the involvement of hypercoagulability in the pathogenesis of type 2 diabetes.

**Supplementary Information:**

The online version contains supplementary material available at 10.1007/s10654-024-01162-0.

## Introduction

Coagulation factors are proteins involved in blood clot formation, working collaboratively to control blood clotting. Although activation of the coagulation system is essential to prevent excessive bleeding in damaged blood vessels, abnormal increases in coagulation factor levels result in an undesirable hypercoagulable state. Hypercoagulability has been observed in individuals with impaired glucose metabolism, endothelial dysfunction, and type 2 diabetes [1–7]. Prior research demonstrated that hyperglycemia contributes to increased levels of coagulation factors leading to hypercoagulability [1–4]. Additionally, our previous study highlighted the association between a higher perfused boundary region indicating a poorer endothelial glycocalyx status and hypercoagulability [5].

Previous studies explored the association between a subset of markers related to coagulation and the incidence of type 2 diabetes, showing that coagulation factor (F) VIII, plasminogen activator inhibitor-1, and activated partial thromboplastin time were associated with the risk of type 2 diabetes after adjustment for known major risk factors of type 2 diabetes [8–13]. However, despite previous observations of hypercoagulability in high-risk populations for type 2 diabetes, the link between hypercoagulability and the incidence of type 2 diabetes is incompletely understood. This underscores the necessity for further research into the association between procoagulant factors and the incidence of type 2 diabetes. In addition, because previous studies mainly focused on individual coagulation factor levels, little is known about the link between the global dynamics of the coagulation system and the incidence of type 2 diabetes. Moreover, additional studies are needed to investigate the mechanism by which coagulation parameters increase the risk of type 2 diabetes.

Glycoprotein acetylation (GlycA), as a marker of systemic inflammation, has been associated with the risk of type 2 diabetes [14, 15]. GlycA is mainly influenced by glycoproteins such as α1-acid glycoprotein, haptoglobin, and α1-antitrypsin [16]. Given that many procoagulants are glycoproteins, GlycA may serve as a plausible mediator for the association between coagulation factors and the incidence of type 2 diabetes. In the current study, we investigated the associations between the levels of FVIII, FIX, FXI, and fibrinogen as well as parameters of the thrombin generation potential that represents the dynamics of global coagulation system and the incidence of type 2 diabetes in the Netherlands Epidemiology of Obesity (NEO) study. In addition, we aimed to explain the underlying mechanism by investigating whether GlycA mediates the effect of these coagulation parameters on the incidence of type 2 diabetes.

## Research design and methods

### Study design and study population

This study was performed in a population-based prospective cohort study, the NEO study. All participants gave their written informed consent. The NEO study was approved by the Medical Ethics Committee of the Leiden University Medical Center (LUMC), Leiden, the Netherlands. The study design and population of the NEO study have been described previously [17]. Briefly, the NEO study was designed to investigate the mechanism that leads to obesity-related diseases. Men and women aged between 45 and 65 years at baseline (September 2008 to September 2012) with a self-reported body mass index (BMI) of 27 kg/m^2^ or higher living in the greater part of Leiden were invited to participate in the NEO study. In addition, all inhabitants aged between 45 and 65 years from one municipality (Leiderdorp) were invited, irrespective of their BMI. Participants were invited for a baseline visit at the NEO study center of the LUMC after an overnight fast. Before the baseline visit, participants completed questionnaires at home to report demographic, lifestyle, and clinical information. At baseline visit, all participants underwent an extensive physical examination including anthropometry and blood sampling. Research nurses recorded names and dosages of current medication used in the month preceding the study visit. After the baseline visit, participants were followed for the occurrence of type 2 diabetes.

**Fig. 1:**
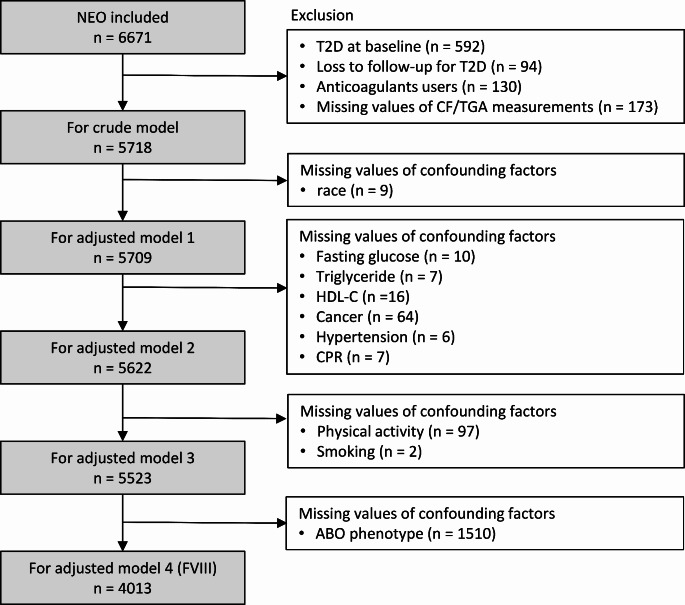
Flow chart of participants inclusion in the present study Abbreviations: NEO: the Netherlands Epidemiology of Obesity study; FVIII: coagulation factor VIII

Participants who had been diagnosed with type 2 diabetes before or at the baseline visit (*n* = 592), or those without any follow-up information regarding the incidence of type 2 diabetes (*n* = 94) were excluded for the present study. We also excluded participants who used vitamin K antagonists or heparin at the baseline visit (*n* = 131) as these medications may influence coagulation factor levels and thrombin generation potential. As direct oral anticoagulants (DOACs) were not commonly used in the Netherlands during the NEO baseline recruitment, the NEO study did not collect information for the use of DOACs. We additionally excluded participants with missing data on any of the exposures, the mediator, and confounders (*n* = 444). In the analyses involving ABO blood group, participants with missing data for ABO blood group were additionally excluded (*n* = 1510). Figure 1 shows the selection of participants for the present study.

### Data collection

#### Hemostatic factors and thrombin generation parameters

Coagulation factor levels and parameters of thrombin generation potential were measured from fasting blood samples collected at the baseline visit. Fasting blood samples were drawn into tubes containing 0.106 M trisodium citrate (Sarstedt, Etten-Leur, the Netherlands). FVIII, FIX, and FXI activity were measured using a factor-specific clotting assay with ACL TOP 700 analyzer (Werfen, Barcelona, Spain). Fibrinogen levels were measured by the method of Clauss [18]. A thrombin generation assay was performed using the Calibrated Automated Thrombogram^®^ (Diagnostica Stago, Asinères, France) following the instructions of the manufacturer [19]. In brief, platelet-poor plasma samples were added to the assay reagent including phospholipids and a very low tissue factor concentration (86194,TS31.00, STAGO, France). A Fluoroskan Ascent fluorometer (Thermo Scientific, Waltham, MA, USA) detected the fluorescent signal to represent thrombin generation. Five thrombin generation parameters were estimated using the Thrombinoscope software (Thrombinoscope BV, Maastricht, the Netherlands). Lag time measured in minutes indicates the time from induction to the initial thrombin generation. Endogenous thrombin potential (ETP) corresponds to a measure of the total amount of thrombin generation, corresponding to the area under the curve of thrombin generation curve. Peak height measured in nM represents the highest point of thrombin level. Time-to-peak measured in minutes corresponds to the time from the induction to the peak. Velocity indicates how fast thrombin levels rise from the initial point of thrombin generation to the peak height.

#### Incidence of type 2 diabetes

New diagnoses of type 2 diabetes were extracted between October 2017 and July 2018 from the electronic health records of the general practitioners (GP) of the participants. Data from the first extraction (July 2012- November 2013) were added to this extraction [20]. Details of the extraction process are provided in the Online supplemental method. Briefly, health records were screened for the International Classification of Primary Care (ICPC) code T90 (diabetes mellitus) or T90.02 (diabetes mellitus type 2) and the prescription of specific medication corresponding to the Anatomical Therapeutic Chemical code A10 (drugs used in diabetes). The medication list of participants was also checked for the use of insulin, metformin, and sulfonylurea derivatives (including search terms for brand names and abbreviations). The index date was defined by the first date of an ICPC-coded diagnosis, or the first date of prescription of anti-diabetic medication. In case of uncertainty, we checked for a preceding diagnosis of disturbed glucose tolerance (ICPC code A91.5), high glucose concentration data in any lab record (if available) and we read the free text in the health records for signs of diabetes. These findings were discussed by the NEO study adjudication committee to decide on a diagnosis. If the diagnosis remained uncertain, the GP of the participants was contacted to confirm the date and diagnosis. Time of follow-up was defined as the number of days between the baseline visit and the date of diagnosis, or censoring due to death, loss to follow-up, or the end of the follow-up (extraction date at the GP in 2013 or 2018), whichever came first. For participants without medical records from their GP more than a year between the last contact and data extraction, we additionally defined a minimized follow-up time for sensitivity analyses as the time between the baseline visit and the date of the last contact with GP because it was unknown whether the participants did not visit their GP afterwards or had switched to another GP.

#### Covariates

On the baseline questionnaire, participants reported age, sex, and family and personal medical history. We considered active cancer patients as those who have been diagnosed within 5 years before baseline and have not been medically cured based on self-report. Hypertension was defined as a systolic blood pressure > = 140mmHg or diastolic blood pressure > = 90mmHg or using antihypertensive drugs. Self-identified race was reported in eight categories, which were grouped into white and others. Participants reported the frequency and duration of their physical activity during leisure time over the past 4 weeks on the Short Questionnaire to Assess Health-enhancing activity, which we expressed in MET-hours per week. Tobacco smoking was reported in three categories with current smoker, former smoker, and never smoker. In women, we grouped the use of contraceptives and hormone replacement therapy into current and past or never users. Menopausal status was classified as premenopausal and perimenopausal or postmenopausal, according to information on oophorectomy, hysterectomy, and self-reported state of menopause in the questionnaire. BMI was calculated by dividing the weight in kilograms by the height in meters squared measured during physical examination. Fasting serum glucose and triglyceride concentrations were measured with enzymatic calorimetric assays (Roche Modular P800 Analyzer, Roche Diagnostics, Mannheim, Germany) and fasting serum high-density lipoprotein cholesterol (HDL-C) concentrations with third-generation homogenous HDL‐C methods (Roche Modular P800 Analyzer, Roche Diagnostics, Mannheim, Germany). ABO blood group was determined by the four *ABO* genetic variants (rs8176719:insC, rs7853989:G > C, rs8176749:G > A, rs8176750:delC) using the imputed genotype data in the NEO study, which has been described in details before [21]. The serum concentrations of C-reactive protein (CRP) were determined using a high-sensitivity CRP assay (TINA-Quant CRP HS system and Modular P800; Roche) [22]. Glycoprotein acetyl (GlycA) concentrations, as the potential mediator, were measured in plasma samples using the Nightingale high throughput NMR metabolomics platform [23].

### Statistical analysis

Baseline characteristics of the study population are presented as absolute numbers and percentages for categorical variables, and continuous variables as medians with interquartile range (IQR) or means with standard deviation (SD). The cumulative incidence of type 2 diabetes was calculated taking the competing risk of death into account. Incidence rates of type 2 diabetes per 1000 person-years with 95% confidence intervals (CI) were calculated for quartiles of coagulation factor levels and thrombin generation parameters. We used Cox proportional hazards regression to calculate hazard ratios (HR) and 95% CI as estimates of the association between the levels of coagulation factor and thrombin generation parameters and the incidence of type 2 diabetes. We adjusted the crude model for age, sex, and race in model 1. In model 2, BMI, baseline levels of fasting glucose, triglyceride, CRP, HDL-C, active cancer, and hypertension were further added, which were expected to have confounding effects. In model 3, we added physical activity, smoking, menopausal status, oral contraceptive use, and hormone replacement which likely have confounding effects. Specifically for the association between FVIII levels and type 2 diabetes, ABO blood group was added to the model 3. In model 4, we added GlycA to examine the mediation effect on the association between coagulation factor levels as well as thrombin generation parameters and the incidence of type 2 diabetes. The proportion of mediation was estimated via R package “CMAverse” with bootstrap 95% CI and *p*-values [24]. The proportional hazards assumption was checked by the Schoenfeld residuals test (Supplemental Figs. 1–5). For models in which the proportional hazard assumption was violated, we added an interaction term between time categorized in periods of 0.5 years and the variable for which the proportional hazard assumption was not met to the model. All analyses were performed using the coagulation factors and thrombin generation parameters as continuous exposure and repeated after categorizing exposures in four groups using their quartiles, using the first quartile as the reference except for lag time and time-to-peak for which we used the fourth quartile as the reference.

In addition, stratified analyses, and sensitivity analyses were performed. First, we stratified all analyses by sex. Second, we aimed to stratify all analyses by BMI of 27 kg/m^2^ (the cut-off value for overweight in the original NEO study). Due to the limited number of participants with BMI less than 27 kg/m^2^, analyses were performed only participants with BMI of 27 kg/m^2^ or higher. In addition, we also performed several sensitivity analyses. First, the follow-up was restricted to the date of the last contact with GP for participants who lacked information in their medical records for more than a year between the last contact and data extraction. Second, we performed a landmark analysis to avoid reverse causation by including only participants who were still in the study after one year. Third, as GlycA is a marker of systemic inflammation, we excluded participants (*n* = 72) with self-reported autoimmune diseases at baseline (i.e., systemic lupus erythematosus, rheumatoid arthritis, and psoriatic arthritis). Lastly, we calculated E-values based on the effect estimates and confidence intervals to investigate the potential impact of unmeasured confounding [25]. All statistical analyses were performed using R version 4.3.0.

### Data and resource availability

The data that support the findings of this study are not openly available due to the privacy of the participants of the NEO study and legal reasons (NEO study participants did not sign informed consent to make their data publicly available). The data will be made available upon request to interested qualified researchers. Data requests should be sent to the NEO Executive Board, which can be contacted via https://www.lumc.nl/org/neo-studie/contact/. Scripts for the analysis are available upon request to the authors.

## Results

The baseline characteristics of the 5718 study participants are provided in Table 1. The mean age of the participants was 56 (SD 6) years and 3051 (53%) were women of whom 2516 (83%) were peri- or post-menopausal. The mean BMI was 29.7 (SD 4.7) kg/m^2^. The medians with lower and upper quartiles of each coagulation factor and thrombin generation parameter are shown in Table 1.

**Table 1:** Baseline characteristics of 5718 middle-aged participants without pre-existing type 2 diabetes in the NEO study

	Study population (*n* = 5718)
**Demographic**	
Age (years)	56 (SD 6)
Women (% women)	3051 (53%)
Race (% white)*	5437 (95%)
**Lifestyle**	
Physical activity (MET/week)*	27.5 (14.0–47.5)
Smoking (% current)*	919 (16%)
**Clinical characteristics**	
Body Mass Index (kg/m2)	29.73 (SD 4.68)
Fasting glucose (mmol/L)*	5.44 (5.11–5.84)
Triglyceride (mmol/L)*	1.21 (0.86–1.71)
HDL-C (mmol/L)*	1.45 (SD 0.41)
C-reactive protein (mg/L)*	1.63 (0.84–3.21)
Cancer (% yes)*	43 (0.8%)
Hypertension (% yes)*	3048 (53%)
Peri or post menopausal (%)	2516 (83%)
Oral contraceptive user (%)	186 (6%)
Hormone replacement therapy (%)	80 (3%)
**Coagulation factors**	
FVIII activity (%)	120 (101–142)
FIX activity (%)	121 (108–134)
FXI activity (%)	118 (105–132)
Fibrinogen (mg/dL)	297 (263–336)
**Thrombin generation parameters**	
Lag time (min)	7.0 (6.2–8.0)
ETP (nM.min)	1126 (883–1388)
Peak height (nM)	80 (61–104)
Time-to-Peak (min)	15.0 (13.8–16.4)
Velocity (nM/min)	10.0 (7.2–14.1)

Data are expressed as mean with standard deviation, median with interquartile ranges or numbers with percentages

Menopausal status, oral contraceptive user, and hormone replacement therapy were calculated in women

Abbreviations: HDL-C: high-density lipoprotein cholesterol; ETP: endogenous thrombin potential

*In order from the top, there are missing values in 9, 100, 4, 10, 7, 7, 6, 66 and 6 participants

The median follow-up time was 6.7 years (IQR 5.9–7.9), and the total follow-up time of the participants was 37,725 person-years. During the follow-up 281 incident diagnoses of type 2 diabetes were identified. When we analyzed the exposures as continuous variables, four coagulation factors and all thrombin generation parameters were associated with the incidence of type 2 diabetes in the crude model (Supplemental Table 1). The effect estimates remained similar after adjustment for all confounders (model 3) except for lag time and time-to-peak for which the effect was attenuated.

**Fig. 2:**
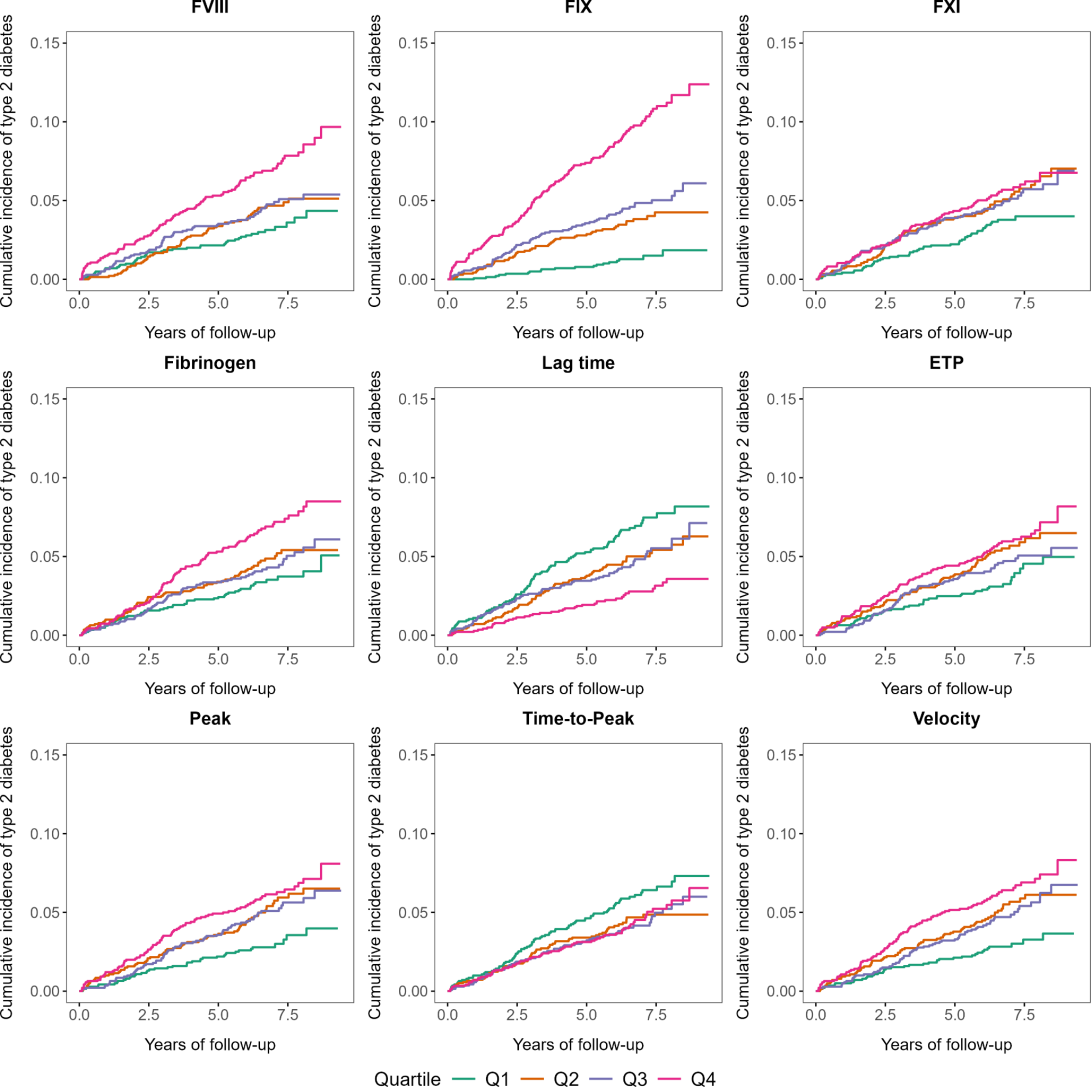
Cumulative incidence plot for type 2 diabetes across the quartile of coagulation factor levels and thrombin generation parameters (*n* = 5718)

When we analyzed the exposures by quartiles, the cumulative incidence of type 2 diabetes increased with the quartiles of procoagulant factor levels and thrombin generation parameters in the crude model (Fig. 2). Incidence rates and HRs for incident type 2 diabetes in each quartile of coagulation factor levels and thrombin generation parameters are shown in Table 2. In the crude model, incidence rates and HRs increased with the quartiles of all coagulation factors and the parameters of thrombin generation potential. Results were similar after adjustment for age, sex, and race in model 1. In adjusted model 2, strong associations were seen for FIX levels with HR of 2.51 (95% CI 1.50–4.20) in the highest quartile compared with the first quartile. Also, thrombin generation parameters ETP, peak, and velocity showed strong associations with the risk of type 2 diabetes We observed weak to no association between FVIII, FXI, fibrinogen, lag time and time-to-peak and the hazard of type 2 diabetes. In the fully adjusted model 3, only marginal changes were observed. Further adjusting for ABO blood group in the association between FVIII levels and the risk of type 2 diabetes resulted in similar effect estimates. Compared with the lowest quartile, the HR (95% CI) of the highest quartile was 2.47 (1.48–4.14) for FIX, 1.37 (0.85–2.20) for FVIII, 1.11 (0.76–1.63) for FXI, 0.98 (0.65–1.48) for fibrinogen, 1.56 (1.07–2.28) for ETP, 1.84 (1.23–2.74) for peak, 1.59 (1.08–2.33) for velocity, 0.92 (0.62–1.38) for lag time, and 1.21 (0.86–1.70) for time-to-peak.

**Table 2:** Incidence rates and hazard ratios for type 2 diabetes according to quartile of coagulation factors and thrombin generation parameters

	Events, *N*	PY	Rate (95% CI)	HR (95% CI)								
				Crude model		Model 1		Model 2		Model 3		Model 4
**FVIII**												
Q1	46	9416.19	4.89 (3.66–6.52)	1		1		1		1		1
Q2	65	9505.18	6.84 (5.37–8.71)	1.40 (0.96–2.04)		1.39 (0.95–2.02)		1.10 (0.75–1.62)		1.21 (0.78–1.88)		1.19 (0.76–1.85)
Q3	66	9395.54	7.02 (5.52–8.93)	1.44 (0.99–2.10)		1.43 (0.98–2.08)		0.95 (0.64–1.41)		1.29 (0.82–2.03)		1.30 (0.82–2.04)
Q4	104	9407.95	11.05 (9.13–13.38)	2.27 (1.60–3.21)		2.27 (1.60–3.22)		1.24 (0.86–1.81)		1.37 (0.85–2.20)		1.31 (0.82–2.11)
**FIX**												
Q1	19	9629.53	1.97 (1.26–3.09)	1		1		1		1		1
Q2	51	9341.42	5.46 (4.15–7.18)	2.77 (1.64–4.70)		2.73 (1.61–4.63)		1.50 (0.88–2.58)		1.46 (0.85–2.51)		1.44 (0.84–2.48)
Q3	73	9767.95	7.47 (5.95–9.39)	3.80 (2.29–6.30)		3.73 (2.25–6.19)		1.81 (1.08–3.02)		1.69 (1.01–2.84)		1.65 (0.98–2.77)
Q4	138	8985.96	15.36 (13.01–18.12)	7.81 (4.83–12.62)		8.11 (5.02–13.12)		2.51 (1.50–4.20)		2.47 (1.48–4.14)		2.33 (1.38–3.93)
**FXI**												
Q1	51	9479.25	5.38 (4.09–7.07)	1		1		1		1		1
Q2	79	9774.34	8.08 (6.49–10.07)	1.50 (1.06–2.14)		1.62 (1.14–2.31)		1.27 (0.89–1.83)		1.35 (0.94–1.95)		1.33 (0.92–1.92)
Q3	71	9367.50	7.58 (6.01–9.56)	1.41 (0.98–2.02)		1.59 (1.11–2.30)		1.28 (0.88–1.86)		1.26 (0.87–1.83)		1.19 (0.81–1.73)
Q4	80	9103.76	8.79 (7.07–10.93)	1.63 (1.15–2.32)		1.97 (1.37–2.83)		1.21 (0.83–1.76)		1.11 (0.76–1.63)		1.02 (0.85–1.50)
**Fibrinogen**												
Q1	51	9780.95	5.21 (3.97–6.86)	1		1		1		1		1
Q2	67	9506.68	7.05 (5.55–8.95)	1.35 (0.94–1.95)		1.41 (0.97–2.03)		0.95 (0.65–1.39)		0.93 (0.63–1.37)		0.89 (0.60–1.32)
Q3	65	9324.61	6.97 (5.47–8.88)	1.34 (0.93–1.93)		1.44 (0.99–2.08)		1.05 (0.72–1.54)		1.04 (0.71–1.54)		1.01 (0.68–1.50)
Q4	98	9112.62	10.75 (8.83–13.09)	2.06 (1.47–2.89)		2.26 (1.60–3.20)		0.96 (0.64–1.43)		0.98 (0.65–1.48)		0.89 (0.58–1.37)
**Lag time**												
Q4	96	8967.09	10.71 (8.77–13.06)	1		1		1		1		1
Q3	73	9500.82	7.68 (6.11–9.66)	0.72 (0.53–0.97)		0.72 (0.53–0.98)		1.05 (0.76–1.44)		1.04 (0.76–1.44)		1.04 (0.75–1.43)
Q2	71	9461.14	7.5 (5.95–9.46)	0.70 (0.52–0.95)		0.72 (0.53–0.98)		1.09 (0.79–1.50)		1.07 (0.77–1.48)		1.10 (0.79–1.54)
Q1	41	9795.80	4.19 (3.08–5.68)	0.39 (0.27–0.57)		0.41 (0.28–0.59)		0.90 (0.61–1.34)		0.92 (0.62–1.38)		0.96 (0.64–1.44)
**ETP**												
Q1	51	9270.43	5.5 (4.18–7.23)	1		1		1		1		1
Q2	75	9337.66	8.03 (6.41–10.06)	1.46 (1.02–2.09)		1.44 (1.01–2.06)		1.54 (1.06–2.25)		1.62 (1.11–2.38)		1.63 (1.11–2.39)
Q3	69	9513.26	7.25 (5.73–9.18)	1.32 (0.92–1.90)		1.31 (0.91–1.88)		1.30 (0.89–1.90)		1.32 (0.90–1.95)		1.32 (0.90–1.95)
Q4	86	9603.50	8.96 (7.26–11.05)	1.63 (1.15–2.31)		1.70 (1.20–2.41)		1.48 (1.02–2.16)		1.56 (1.07–2.28)		1.56 (1.06–2.28)
**Peak**												
Q1	43	9313.05	4.62 (3.43–6.22)	1		1		1		1		1
Q2	75	9396.36	7.98 (6.37-10)	1.73 (1.19–2.52)		1.70 (1.17–2.47)		1.71 (1.15–2.54)		1.79 (1.19–2.68)		1.78 (1.19–2.66)
Q3	73	9488.90	7.69 (6.12–9.67)	1.67 (1.14–2.43)		1.63 (1.12–2.38)		1.57 (1.05–2.35)		1.65 (1.09–2.48)		1.67 (1.11–2.51)
Q4	90	9526.55	9.45 (7.69–11.6)	2.05 (1.42–2.95)		2.09 (1.45–3.01)		1.73 (1.17–2.56)		1.84 (1.23–2.74)		1.81 (1.21–2.71)
**Time-to-Peak**												
Q4	88	9380.03	9.38 (7.62–11.55)	1		1		1		1		1
Q3	62	9288.35	6.68 (5.21–8.55)	0.71 (0.51–0.99)		0.73 (0.52–1.01)		1.05 (0.75–1.48)		0.98 (0.70–1.39)		1.03 (0.73–1.45)
Q2	64	9395.29	6.81 (5.34–8.7)	0.73 (0.53-1.00)		0.74 (0.54–1.03)		1.20 (0.86–1.68)		1.17 (0.83–1.64)		1.20 (0.85–1.68)
Q1	67	9661.19	6.93 (5.46–8.8)	0.74 (0.54–1.02)		0.77 (0.56–1.07)		1.22 (0.87–1.69)		1.21 (0.86–1.70)		1.11 (0.78–1.57)
**Velocity**												
Q1	42	9387.67	4.47 (3.31–6.05)	1		1		1		1		1
Q2	74	9457.83	7.82 (6.24–9.82)	1.75 (1.20–2.55)		1.72 (1.18–2.51)		1.27 (0.85–1.88)		1.22 (0.82–1.82)		1.24 (0.83–1.84)
Q3	72	9459.79	7.61 (6.05–9.58)	1.70 (1.16–2.49)		1.66 (1.14–2.44)		1.19 (0.81–1.77)		1.26 (0.85–1.87)		1.27 (0.86–1.89)
Q4	93	9419.57	9.87 (8.07–12.09)	2.21 (1.53–3.18)		2.23 (1.55–3.22)		1.51 (1.03–2.22)		1.59 (1.08–2.33)		1.58 (1.08–2.33)

Incidence rate per 1,000 person-years was calculated in crude model (*n* = 5718)

Model 1: Adjusted for age, sex, and race (*n* = 5709)

Model 2: Adjusted for Model 1 + body mass index, fasting glucose, triglyceride, high-density lipoprotein-cholesterol, c-reactive protein, cancer, and hypertension (*n* = 5622)

Model 3: Adjusted for Model 2 + physical activity, smoking, menopausal status, oral contraceptive use, and hormone replacement therapy (*n* = 5523) as well as ABO blood type for FVIII (*n* = 4013)

Model 4: Adjusted for Model 3 + Glycoprotein acetylation (*n* = 5502 for others and *n* = 4001 for FVIII)

PY, person-years; HR, hazard ratio; CI, confidence interval

To investigate the mediation effect of GlycA on the observed associations, we further added GlycA in model 4. The effect estimates remained similar after addition of GlycA to the model and the proportion mediated was minimal (Supplemental Table 2). These findings suggest the effects of coagulation factor levels and thrombin generation on the incidence of type 2 diabetes were only marginally mediated through GlycA.

In sex-stratified analyses, the association and trends observed were similar; however, the confidence intervals were wider (Supplemental Tables 3 and 4). In individuals with BMI of 27 kg/m^2^ or higher, similar associations were observed as in the complete analysis (Supplemental Table 5). Supplemental Table 6 shows the results from the sensitivity analysis in which we used minimized follow-up time. Supplemental Table 7 presents the results from the landmark analyses using only those who are present in the study after one year. Supplemental Table 8 shows the results after excluding 72 participants with autoimmune disease at baseline. The results in Supplemental Tables 6, 7, and 8 were all similar to the main analysis results in Table 2, suggesting the robustness of the main analysis results. Supplemental Table 9 presents the E-values based on the effect estimates and confidence intervals. For example, the estimated HR (95% CI) was 2.47 (1.48–4.14) for the association between the highest quartile of FIX activity and the incidence of type 2 diabetes. The corresponding E-value of 4.38 means that an unmeasured confounding factor would need to be associated with a HR of 4.38 for the outcome and a RR of 4.38 for the exposure category to explain away the observed estimate. The lower bound E-value was 2.32, showing that unmeasured confounding should also be substantial to shift the lower bound of the CI below 1.

## Discussion

In this cohort study, we investigated the associations between coagulation parameters and incident type 2 diabetes, as well as the mediating role of GlycA in the observed associations. Elevated levels of FIX, as well as prothrombotic parameters of thrombin generation such as peak, velocity, and ETP were associated with an increased incidence of type 2 diabetes after adjustment for confounding factors. The levels of FVIII, FXI, fibrinogen, thrombin generation lag time and time-to-peak were also associated with an increased incidence of type 2 diabetes in the crude model, however, these associations were attenuated after further adjustment for confounding factors. The mediation effect of GlycA on the association between coagulation factor levels, thrombin generation parameters and the incidence of type 2 diabetes was negligible.

The current study shows that elevated FIX levels and enhanced thrombin generation potential are involved in the pathogenesis of type 2 diabetes. Previous studies mainly investigated a subset of procoagulants including FVIII and fibrinogen [9–11]. Little was known about the association between thrombin generation potential that represents global coagulation levels and the incidence of type 2 diabetes. Therefore, we investigated the association between FIX levels, thrombin generation potential, and the incidence of type 2 diabetes in a follow-up study, showing robust associations after adjustment for confounding factors. Fibrinogen levels were consistently not associated with the risk of type 2 diabetes after adjustment for well-known risk factors for type 2 diabetes in literature and our study [9–11]. Duncan and colleagues observed that increased levels of FVIII were associated with an increased risk of type 2 diabetes, but this association was observed solely in women [11]. In the current study, we also observed that FVIII levels showed no to weak association with the incidence of type 2 diabetes in the total population while we found that the HR (95% CI) was 1.99 (0.95–4.17) in women and 0.97 (0.51–1.83) in men when comparing the highest quartile to the lowest quartile. However, the limited number of participants in sex-stratified analysis hinders the interpretation of our findings with sufficient statistical power. Further research may help fully understand whether specific characteristics in women influence the association between FVIII and the risk of type 2 diabetes.

A growing body of evidence has implicated the interplay of thrombosis and inflammation in disease pathogenesis. Inflammation can activate the coagulation cascade, and conversely, coagulation can induce inflammation [26]. As inflammation is proposed as a key underlying mechanism in the pathogenesis of type 2 diabetes, it may distort the effect of hypercoagulability on the incidence of type 2 diabetes. To address the independent associations between the coagulation factors and incident type 2 diabetes, we adjusted the associations for inflammation marker, CRP. Although the effect estimates were attenuated after adjustment for CRP and other risk factors of type 2 diabetes, we still observed the association between FIX as well as thrombin generation parameters, such as peak and velocity, and incident type 2 diabetes, suggesting the independent involvement of these factors in type 2 diabetes.

Impaired glucose metabolism and endothelial dysfunction are two major risk factors leading to type 2 diabetes, and both are also associated with hypercoagulability. In a previous publication in the NEO study, we observed that fasting glucose levels, HbA1c levels, and postprandial glucose concentrations were associated with FVIII, FIX and FXI levels [4]. We also showed the association between higher perfused boundary region (PBR) indicating poorer endothelial glycocalyx status and coagulation factor levels [5]. Similarly, ABO blood group affects both plasma levels of FVIII [27, 28] and the risk of type 2 diabetes [29]. Given the previous evidence, we considered impaired glucose metabolism, endothelial dysfunction, and ABO blood group as potential confounders. After adjustment for fasting blood glucose, the associations between FIX as well as thrombin generation parameters and type 2 diabetes remained. Also, accounting for the influence of ABO blood group resulted in only minimal change of the association between FVIII and type 2 diabetes. Because PBR parameters were measured in a subset of the NEO study population (*n* = 771), we were not able to draw further conclusions by accounting for endothelial dysfunction in the current study. Nevertheless, our results show that hypercoagulation could play a role in the development of type 2 diabetes in addition to well-known risk factors. Further investigation controlling the remaining confounding effects remains to be performed.

Emerging evidence indicates that GlycA levels are associated with the incidence of type 2 diabetes as a marker of systemic inflammation [14, 15]. GlycA signals originate from residues on glycan part of glycoproteins [16]. Considering that many coagulation factors including FVIII, FIX, and fibrinogen are glycoproteins, we hypothesized that GlycA levels could mediate the observed associations in the present study. However, we only observed a negligible mediation effect of GlycA on the association between FIX as well as thrombin generation parameters and incident type 2 diabetes. This finding suggests that the coagulation system could influence the risk of type 2 diabetes through other mechanisms that have yet to be fully characterized. Hypercoagulability is closely related to inflammation, the complement system, and glucose and lipid metabolism, with complex interactions among these factors [26, 30, 31]. Despite including several factors such as CRP, triglycerides, or glucose levels in the present study, it may be insufficient to comprehensively capture the complex relationships. Further investigations on the underlying mechanisms could help explain the effect of increased coagulation parameters on type 2 diabetes.

Although many hemostatic factors and hypercoagulable states are well-established risk factors for cardiovascular diseases, fewer studies have explored the connection between hypercoagulation and the risk of type 2 diabetes. An increasing body of evidence highlights shared pathophysiological pathways underlying cardiovascular and metabolic diseases, collectively termed “cardiometabolic diseases”. Our study reinforces this link by suggesting that hypercoagulation may elevate the risk of type 2 diabetes in a general Dutch population. Given the strong relationship between cardiovascular disease and type 2 diabetes, our findings may suggest a new perspective on mitigating the risk of both conditions through anticoagulant therapy. To our knowledge, no previous study has investigated the potential beneficial effects of anticoagulants on type 2 diabetes risk in a general population. However, due to concerns about bleeding risks, anticoagulants are unlikely to be repurposed as a preventive strategy for type 2 diabetes. Nevertheless, since thrombotic disorders and type 2 diabetes are often concomitant, and anticoagulants are commonly prescribed to individuals with a high risk of thrombotic disease, treatment with anticoagulants may help prevent comorbid type 2 diabetes for patients with thrombotic disorders. Future research is still needed to investigate this potential impact.

The strength of our study is that we investigated the association between coagulation factor levels as well as parameters of thrombin generation and the incidence of type 2 diabetes in a large longitudinal cohort. Furthermore, we studied individual coagulation factor levels as well as global coagulation markers by measuring the endogenous thrombin generation potential. In addition, we tried to explain the mechanism by which the coagulation system influences the risk of type 2 diabetes via GlycA. However, the present study also has several limitations. First, because the NEO study included participants aged between 45 and 65 years, with an oversampling of overweight and obese individuals, it is warranted to replicate our study in diverse populations to validate and generalize our findings. Second, although we included a large set of potential confounders in the model, residual confounding may remain. To address the impact of unmeasured confounding, we calculated E-values for the estimated HRs. Considering we included a large set of potential confounders in the model, the likelihood of any remaining unmeasured confounders with such a strong effect is low. Third, we performed a complete case analysis which might cause invalid estimation due to selection bias and compromised statistical power and precision. However, as the number of missing values was limited in the present study (e.g., 2.6% missing for exposures), the complete case analysis is unlikely to have a meaningful impact [32]. Fourth. because inflammation and the coagulation system are closely related, it is difficult to disentangle whether inflammation is a mediator or a confounder in the association between coagulation factors and the incidence of type 2 diabetes. Thus, adjusting for these inflammatory markers might lead to underestimation of the associations. Last, in the stratified analyses, the sample size was reduced in subgroups, which led to reduced power and wider confidence intervals, limiting the interpretation of hazard ratios in these subgroups.

In conclusion, elevated levels of FIX and parameters of thrombin generation such as peak, velocity, and ETP were associated with the incidence of type 2 diabetes. Our results suggested the role of hypercoagulability in the development of type 2 diabetes. Further investigations are warranted to identify the specific high-risk group based on coagulation factor levels and disentangle the underlying pathophysiological pathways associated with these coagulation factors, which can potentially lead to the discovery of novel therapeutic targets for the treatment of type 2 diabetes.

## Electronic supplementary material

Below is the link to the electronic supplementary material.

Supplementary Material 1

Supplementary Material 2

## Declarations

### Prior presentation

A part of the results in this article was presented at an Annual Dutch Diabetes Research Meeting 2022 on 03 November 2022 in Wageningen, the Netherlands.
