# Supplementary material for: Associations of coagulation parameters and thrombin generation potential with the incidence of type 2 diabetes: mediating role of glycoprotein acetylation
Source: Eur J Epidemiol. 2024 Oct 15;39(10):1171–81. doi: 10.1007/s10654-024-01162-0 (PMC11599431; doi:10.1007/s10654-024-01162-0)
Supplement: Supplementary file 1 — Supplementary Material 1 [file 10654_2024_1162_MOESM1_ESM.docx]

**Supplemental methods**

Between October 2017 and July 2018 data from electronic health records from the general practitioner of each participant was extracted. Data from a previous extraction done between July 2012 and November 2013 were added.

From the electronic health records we extracted three parts and each part was flagged if an indication for a diagnosis of diabetes mellitus (DM) was present:

1) **Episode list.** This list only contains International Classification of Primary Care (ICPC) codes with a date of entry. The episode list was flagged if ICPC code T90 or T90.2 was reported.

2) **Medication list.** This list contains ICPC codes, Anatomical Therapeutic Chemical (ATC) codes and free text. Each entry is accompanied with a date of prescription. The medication list was flagged if ICPC code T90 or T90.2, or ATC code A10 (Drugs used in diabetes), or a prescription for metformin, insulin, or sulfonylurea derivatives was reported. The text was also searched for synonyms, abbreviations and brand names of glucose-lowering medication (see list below).

3) **Journal list.** This list is a free text filled out during each visit with a date of entry. In this list, ICPC or ATC codes may be provided as well. The journal list was flagged if ICPC code T90 or T90.2 was reported.

Based on these criteria, we identified 864 participants with an indication for DM (both prevalent and incident cases). The index date was defined by the first entry date of an ICPC-coded diagnosis, or the first date of prescription of anti-diabetic medication.

Cases with only a flag in journal, or only in the medication list were checked further. These checks included screening for a previous indication for disturbed glucose tolerance (ICPC A91.5) in any list, lab results on fasting glucose or hb1ac , and all free text in the journal was read to identify any terms related to DM (e.g. glucose control, high blood sugar). Doubtful findings were discussed by the NEO study adjudication committee to verify the diagnosis. If the diagnosis remained inconclusive, the GP of the participant was contacted to confirm the date and diagnosis. If the date of diagnosis was before or within 30 days after the baseline visit, a case was classified as prevalent. If the date of diagnosis was >30 days after baseline visit, a case was classified as incident.

Next, we compared the incident and prevalent cases as extracted from the health records of the GP with baseline DM status based on self-report, fasting plasma glucose ≥7 mmol/L, and use of glucose-lowering medication at baseline. For all discrepancies between baseline DM status and DM status according to the health records, additional checks as above were performed. The GP was contacted if the diagnosis remained inconclusive. The flowchart below provides details and number of cases for the process of the ascertainment of diabetes status.

**Flow chart of diagnoses of diabetes status in the NEO study**


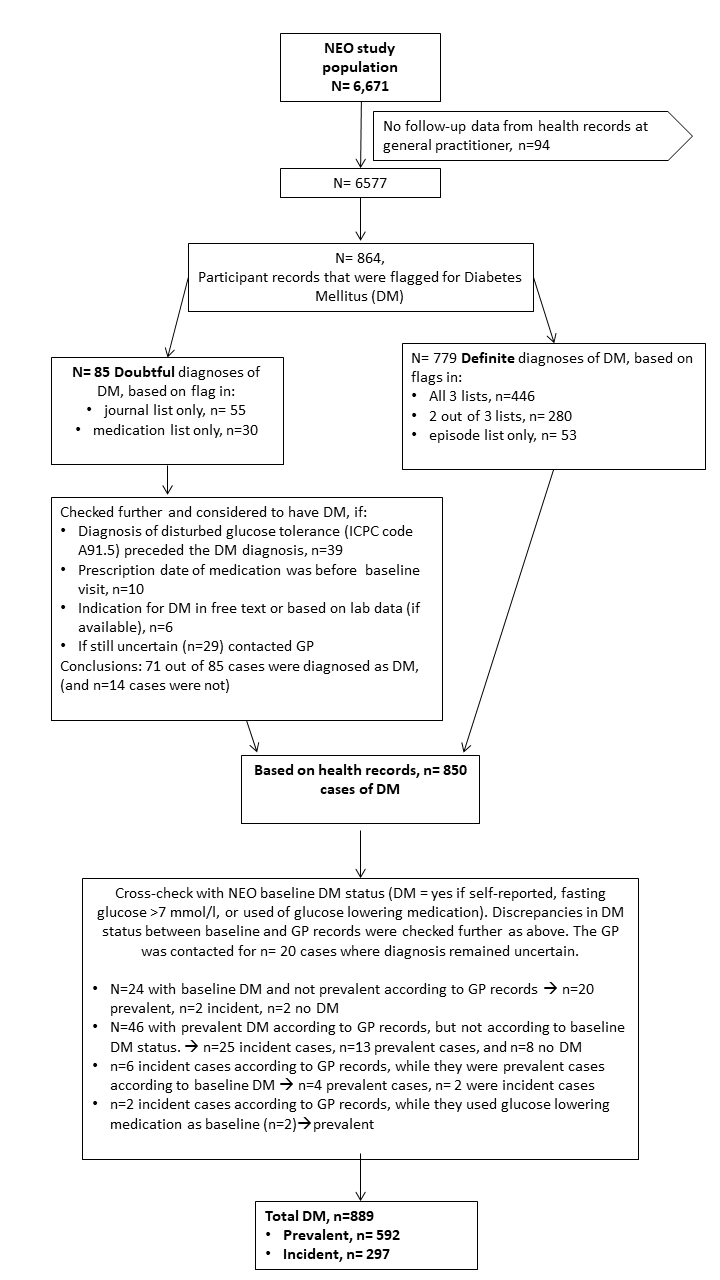


**List of search terms for diabetes medication**

| insulin |
| --- |
| actrapid |
| insuman |
| humuline |
| humalog |
| liprolog |
| novorapid |
| fiasp |
| apidra |
| insulatard |
| protaphane |
| actraphane |
| mixtard |
| novomix |
| ryzodeg |
| abasaglar |
| lantus |
| toujeo |
| semglee |
| levemir |
| tresiba |
| suliqua |
| xultophy |
|  |

| metform |
| --- |
| glucient |
| metnova |
| metfocell |
| yalformet |
| glibencl |
| amglidia |
| tolbutam |
| gliclaz |
| diamicron |
| glimepiride |
| amaryl |
| fertin |
| glucovance |
| avandamet |
| competact |
| glubrava |
| tandemact |
| janumet |
| velmetia |
| efficib |
| ristfor |
| eucreas |
| zomarist |
| icandra |
| incresync |
| komboglyze |
| jentadueto |
| vipdomet |
| xigduo |
| ebymect |
| vokanamet |
| glyxambi |
| synjardy |
| qtern |
| acarbose |
| glucobay |
| miglitol |
| diastabol |
| rosiglit |
| avandia |
| pioglit |
| actos |
| glustin |
| glidipion |
| sitaglip |
| xelevia |
| januvia |
| steglujan |
| tesavel |
| ristaben |
| vildaglip |
| galvus |
| jalra |
| xiliarx |
| saxaglip |
| onglyza |
| aloglip |
| vipidia |
| linaglip |
| trajenta |
| exenat |
| byetta |
| bydureon |
| liraglut |
| victoza |
| saxenda |
| dulaglut |
| trulicity |
| semaglut |
| ozempic |
| dapaglif |
| forxiga |
| edistride |
| canaglif |
| invokana |
| empaglif |
| jardiance |
| repaglin |
| prandin |
| novonorm |
| enyglid |
| nateglin |
| starlix |

Supplemental Figure 1. Schoenfeld residuals diagnostic plots in crude model


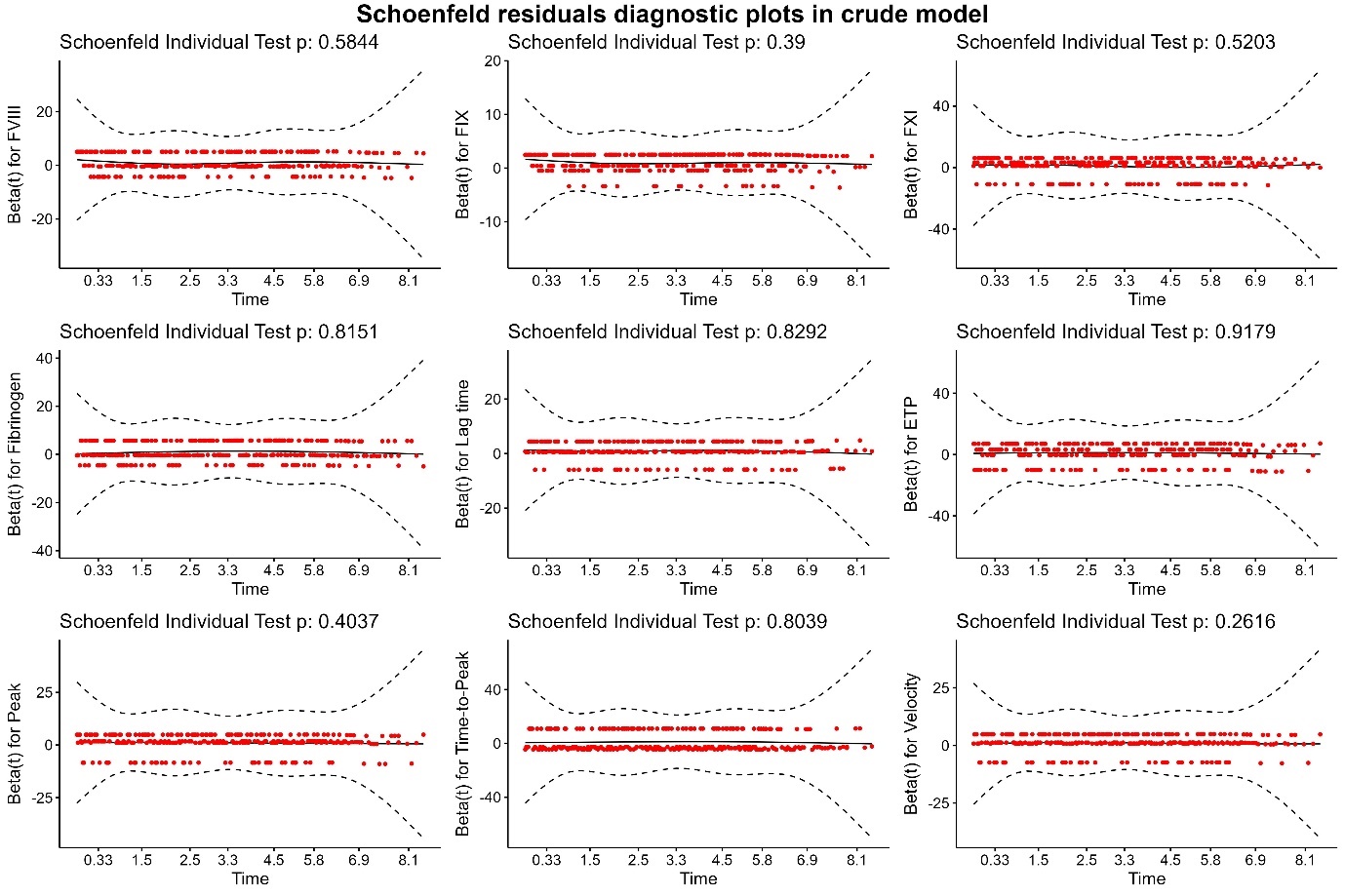


Supplemental Figure 2. Schoenfeld residuals diagnostic plots in model 1


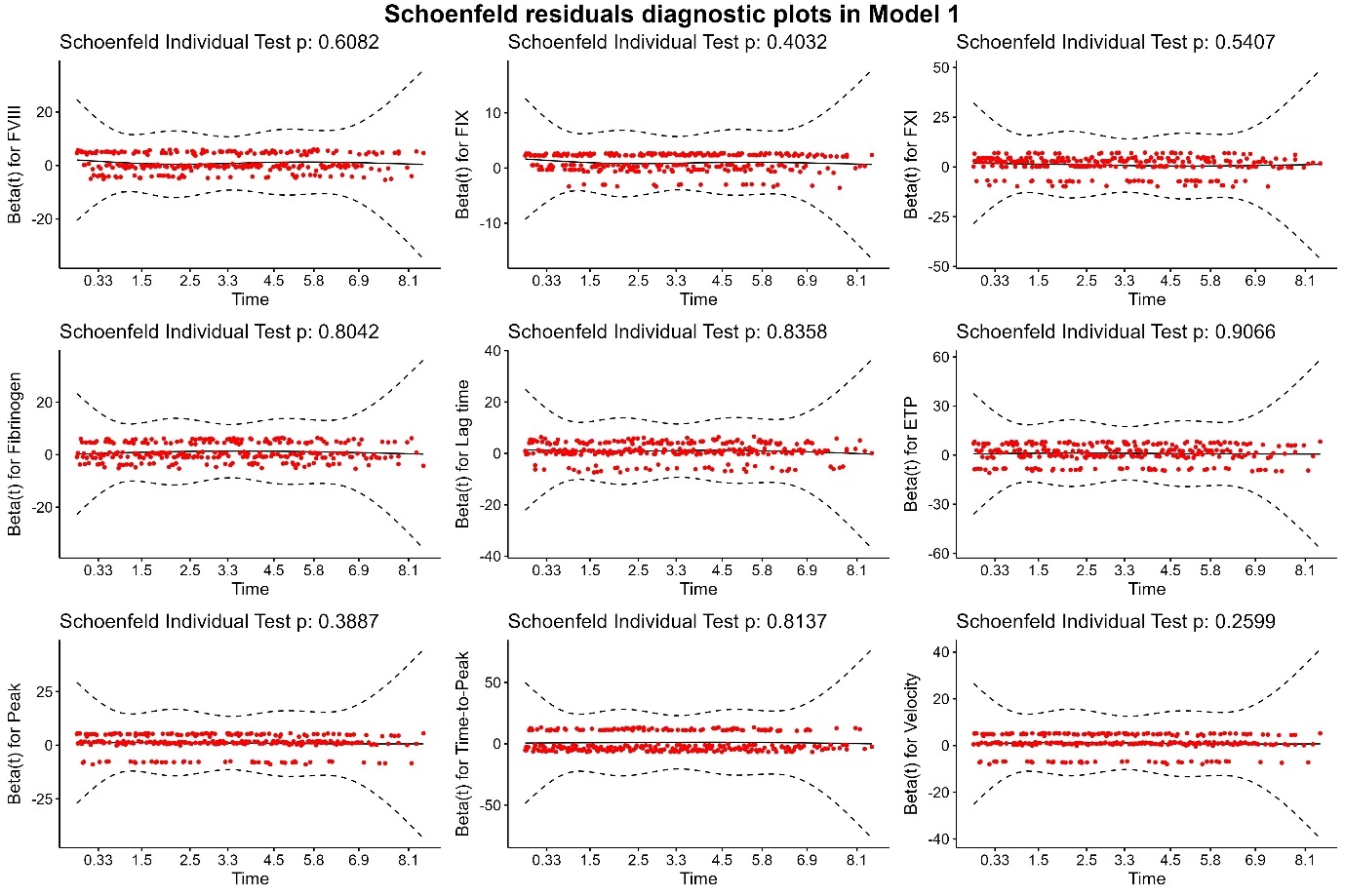


Supplemental Figure 3. Schoenfeld residuals diagnostic plots in model 2


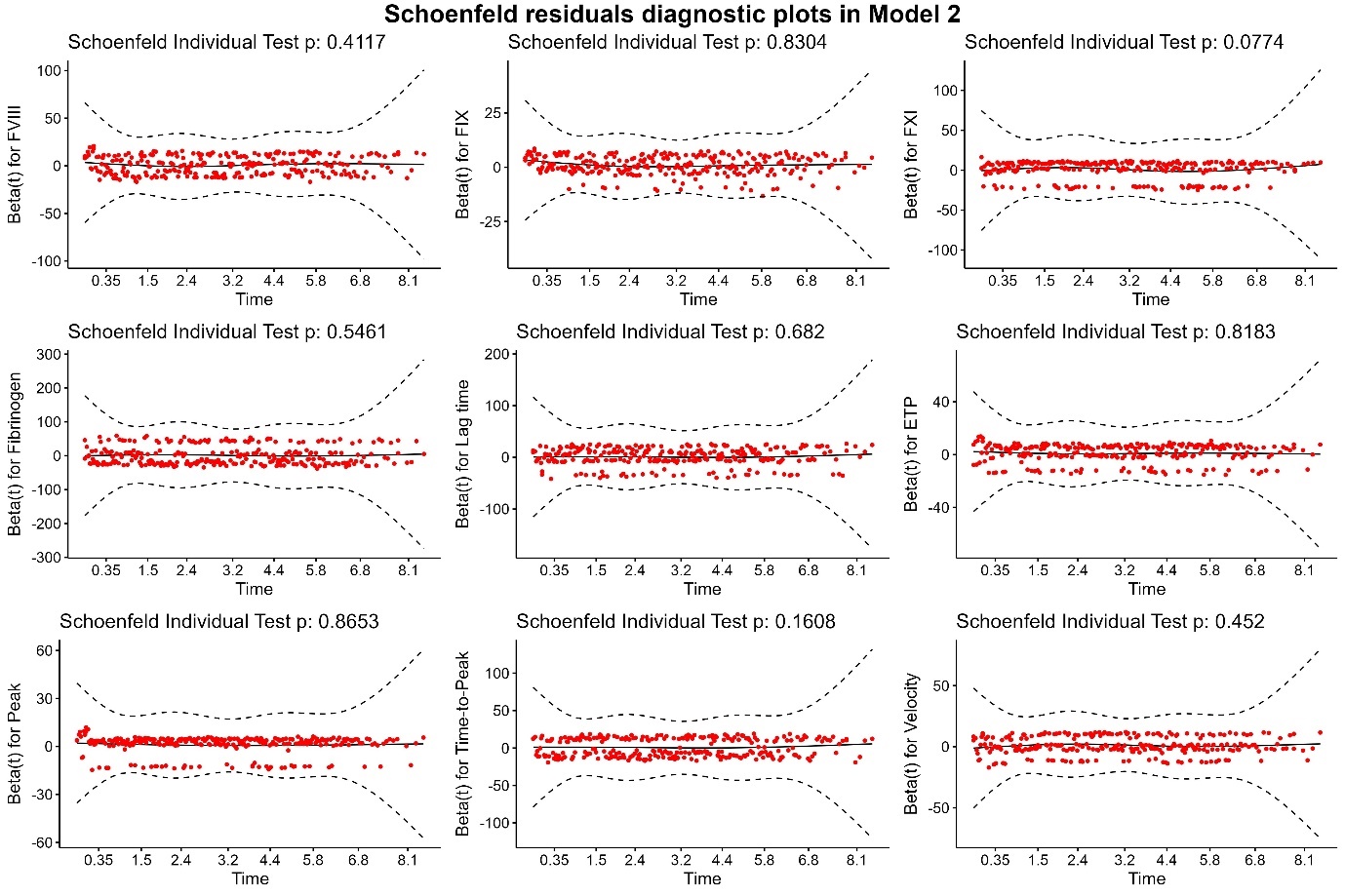


Supplemental Figure 4. Schoenfeld residuals diagnostic plots in model 3


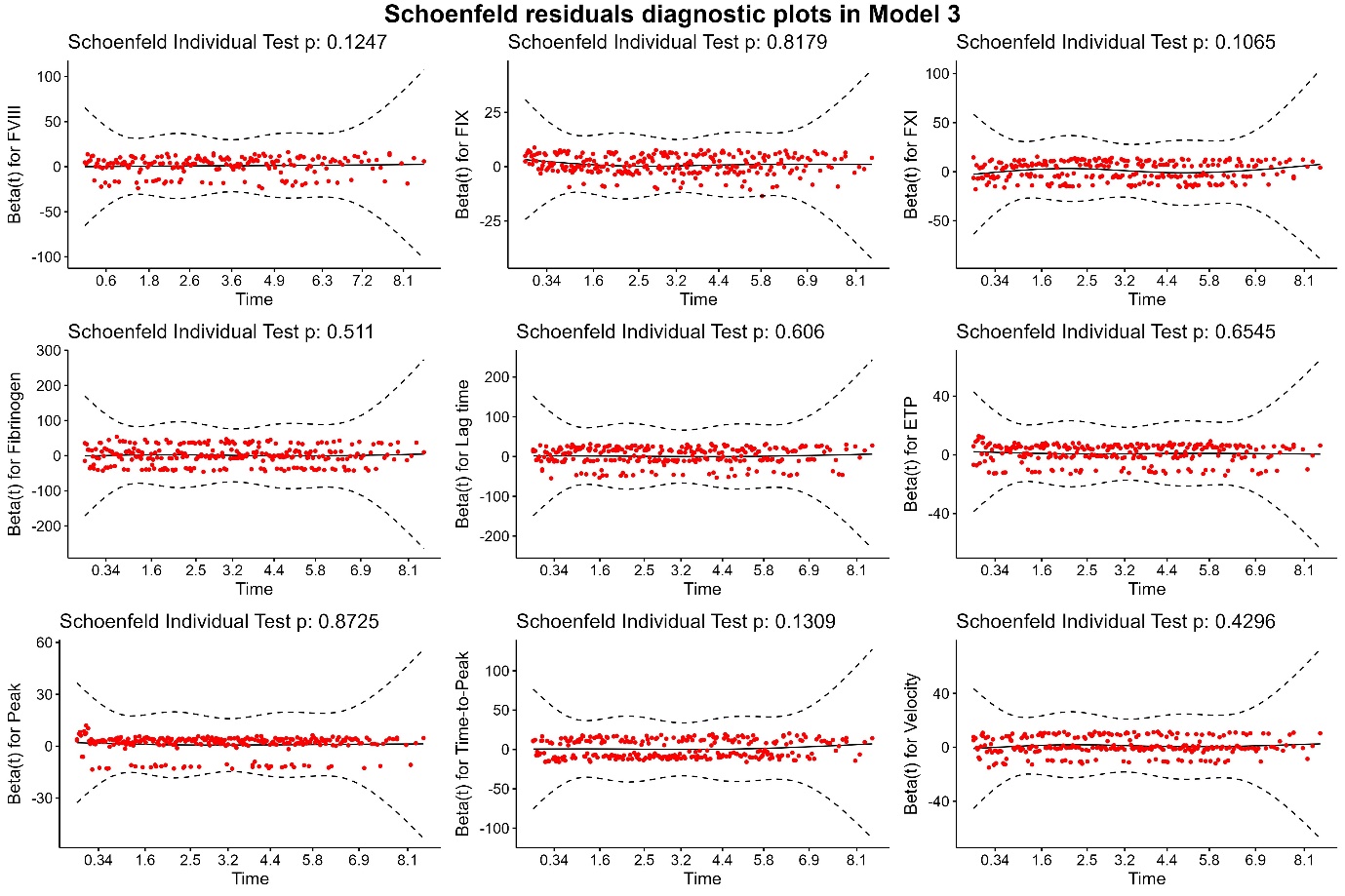


Supplemental Figure 5. Schoenfeld residuals diagnostic plots in model 4


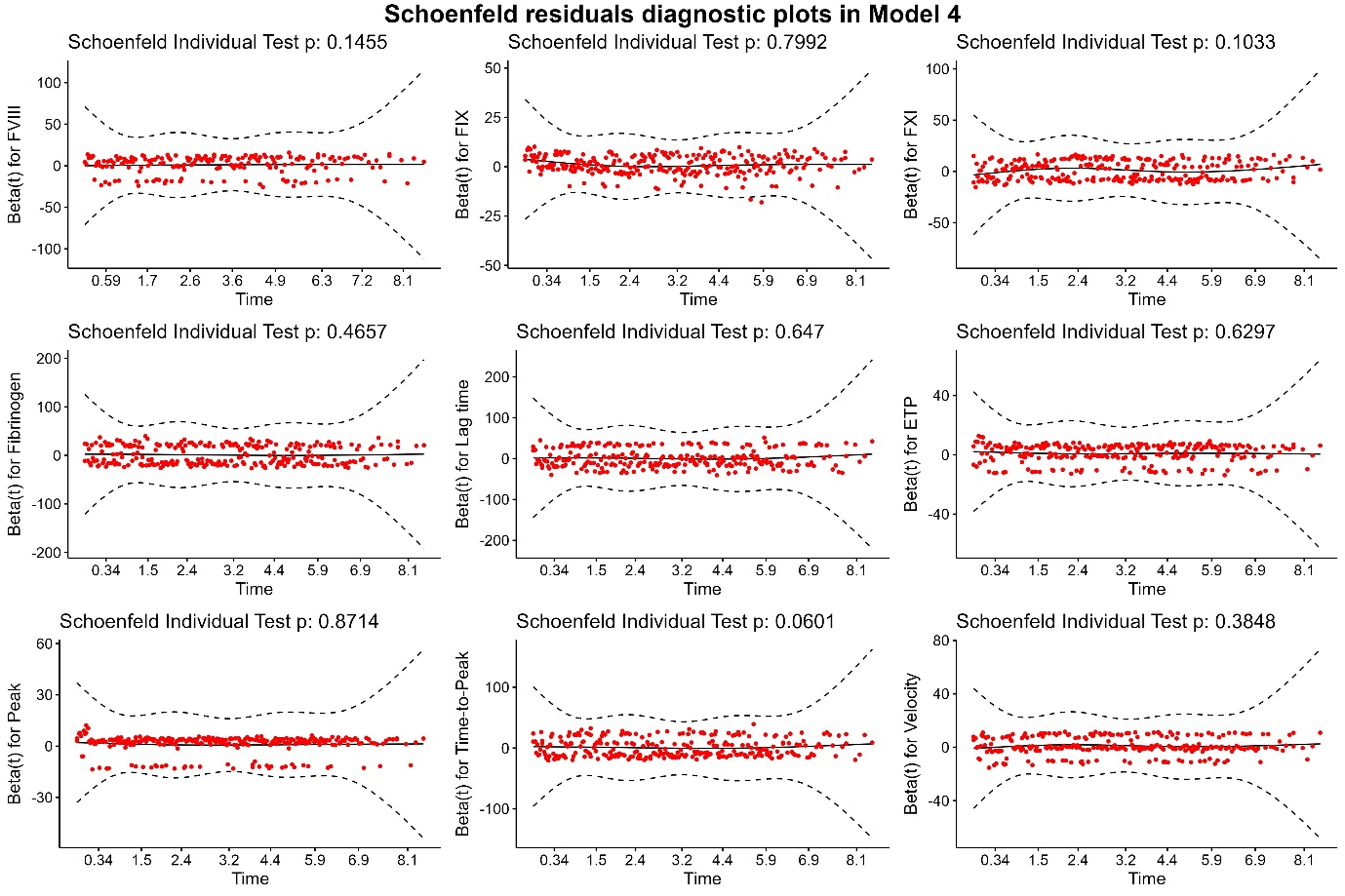


Supplemental Figure 6. Directed acyclic graph


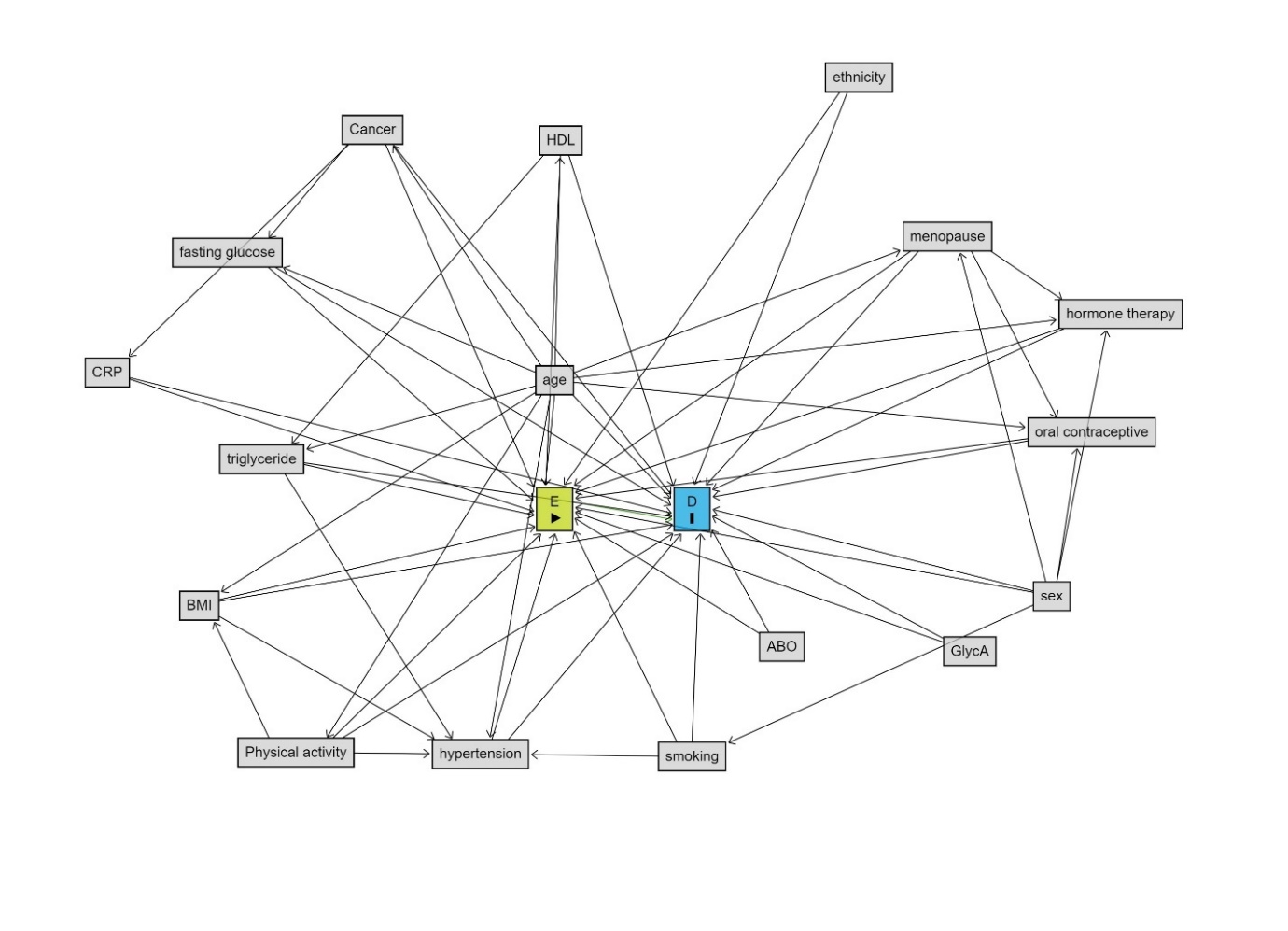


A directed acyclic graph was created via DAGitty (reference: Textor J, van der Zander B, Gilthorpe MS, Liskiewicz M, Ellison GT. Robust causal inference using directed acyclic graphs: the R package 'dagitty'. Int J Epidemiol. 2016 Dec 1;45(6):1887-1894. doi: 10.1093/ije/dyw341. PMID: 28089956.)
